# Neutron spin echo spectroscopy with a moving sample

**DOI:** 10.1038/s41598-023-39854-4

**Published:** 2023-08-11

**Authors:** Manuchar Gvaramia, Philipp Gutfreund, Peter Falus, Antonio Faraone, Michihiro Nagao, Max Wolff

**Affiliations:** 1 Department for Physics and Astronomy, Uppsala University, Regementsvägen 1, SE-75120 Uppsala, Sweden; 2 Institut Laue–Langevin, CS 20156, 38042 Grenoble Cedex 9, France; 3 Center for Neutron Research, National Institute of Standards and Technology, Gaithersburg, MD USA; 4 Department of Materials Science and Engineering, University of Maryland, College Park, MD USA; 5 Department of Physics and Astronomy, University of Delaware, Newark, DE USA

**Keywords:** Colloids, Polymers, Rheology

## Abstract

Neutron spin echo spectroscopy is a high resolution inelastic neutron scattering method probing nanosecond dynamics. It is well suited to study the atomistic motion in polymer systems and contributes to our understanding of viscoelasticity. However, for samples under shear, or moving samples in general, Doppler scattering has to be considered. We compare the measured phase shift and depolarisation due to Doppler scattering from a rotating graphite disk to numerical and analytical calculations and find excellent agreement. This allows to take into account Doppler scattering during the data processing and makes longer Fourier times as well as higher shear rates and Q ranges possible with neutron spin echo spectroscopy, enabling for example the study of polymers under high shear.

## Introduction

The specific properties of neutrons offer several unique features for the study of materials. The fact that the neutron has a rest mass results in a significantly lower energy as compared to photons with nm wavelength. As a result neutrons are an excellent probe for the study of low energy excitations, such as phonons or molecular rotations as well as the study of diffusion via, so called quasi-elastic scattering^1,2^.

Depending on the energy and time scales of interest different scattering methods are available. The best energy resolution or longest time scales are reached on neutron spin echo (NSE) spectrometers and they are best suited to study slow dynamics^3^. In addition to the low energy, the neutron is sensitive to the nucleus and thus isotope exchange allows to introduce contrast in a sample made from the same chemical elements. Another advantage of the nuclear interaction of neutrons with matter is the appearance of incoherent scattering, which allows the investigation of tracer diffusion without having to introduce tracer particles. These facts combined with the excellent energy resolution of NSE made it possible to experimentally verify theories on polymer dynamics, such as the reptation model^4^ and extensions to it, e.g. contour length fluctuations^5^ and constrain release^6^. The complex and slow dynamics of polymers have severe impact on their rheological properties and result in viscoelasticity, e.g. a viscosity that is depending on shear rate. However, till now NSE experiments were almost exclusively performed on samples at rest, while a detailed understanding of the molecular dynamics under shear is required to fully understand viscoelasticity and computer simulations indicate changes in the intermediate scattering function of polymers exposed to high shear rates (Weissenberg number (Wi) larger than 1)^7^.

In contrast to NSE neutron small angle scattering (SANS) is done in a routine manner. Rheo-SANS is a powerful technique that can provide information on both the macroscopic and microscopic behavior of materials. On the macroscopic scale, rheology measurements provide information on the material’s viscoelastic properties, such as its shear modulus and viscosity. On the microscopic scale, SANS provides information on the material’s nanoscopic structure, such as the size and distribution of particles or the conformation and self-assembly of molecular chains. By combining these two techniques, Rheo-SANS can reveal how the microstructural properties of a material influence its macroscopic flow behavior, and vice versa^8^. In rheological experiments shear is often applied either in a Couette or cone-plate geometry. The cone-plate geometry is preferable for high viscosity samples, such as polymer melts.

In case of sample speeds on the order of the neutron speed Doppler scattering may engender a change of the neutron scattering angle as shown in diffraction experiments using a rotating crystal^9^ and SANS studies on aerosol droplets flying parallel to the neutron momentum transfer, *Q*, at the same speed as the neutrons^10^. For typical rheology experiments the sample speed $v_s$ is on the order of m/s and thus significantly slower than the neutron speed $v_n$ of around 300 m/s and changes of the scattering angle are not expected. However, high resolution inelastic neutron scattering is able to detect energy changes on the order of 1% of the neutron energy, or even below that, and is thus sensitive to Doppler scattering at these relatively slow speeds as shown with neutron backscattering^11^ and NSE spectroscopy^12,13^ on sheared liquids and by NSE on moving flux line lattices in a superconductor^14^. To study the molecular dynamics under shear the Doppler scattering has to be known and for quasielastic scattering it was shown that the molecular dynamics can be extracted from the wings of the spectrum^15,16^ irrespective of the Doppler scattering and an anisotropy in the diffusivity of polymer micelles was reported under shear^16,17^.

The excellent energy resolution in NSE^3^ is achieved by a polarized neutron beam guided through two symmetrical magnetic fields before and after interacting with a sample. If the spin of the neutron beam is perpendicular to a magnetic field, it precesses. The total accumulated phase of the neutron spin depends on the neutron velocity/energy and the field integral. After the sample the neutron beam passes a second magnetic field coil, which is identical to the first one but opposite in direction with respect to the neutron spin. Accordingly, the spin will precess back in the second coil and after both coils the initial neutron polarisation is recovered in case of elastic scattering. Note, that this holds for neutrons of all wavelengths and large divergence, which allows using high flux and have good energy resolution at the same time. If, on the other hand, a sample interacts inelastically with the neutron beam, changing the mean speed of the neutrons, the polarisation is not fully recovered. To scan for different energy transfers to the neutron from the sample the two precession coils may be detuned (slight variation of the field integral in the first and second coil) to compensate for the difference in velocity and fully recover the polarisation for a given energy transfer. In order to increase the sensitivity of the technique to a small change in mean neutron velocity, typically large magnetic fields are applied leading to hundred thousands of neutron spin rotations along the flight path. The sensitivity or typical time scale ($\tau$) is scanned by scanning the magnetic field strength in the coils and therefore a depolarization of the beam as a function of typical time scale is measured, which is proportional to the intermediate scattering function. This procedure is similar to other coherent spectroscopy techniques using electromagnetic pulses and recovering its echo after interaction with a sample, as e.g. nuclear magnetic resonance, therefore the name spin-echo.

**Figure 1:**
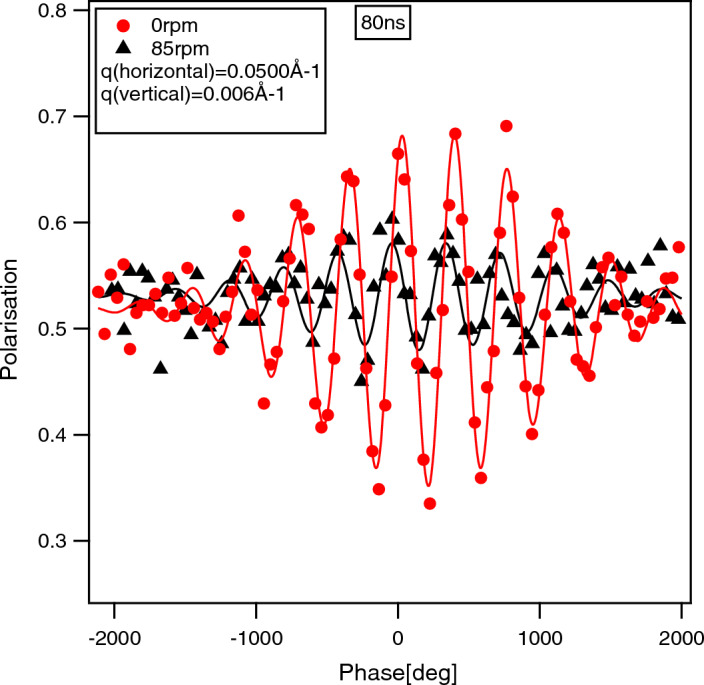
Measured polarisation as a function of tuning coil current, in units of phase rotation, for $\tau$ = 80 ns for a graphite block at rest (red circles) and a rotation speed of 85 rpm (black triangles, corresponding to approx. 0.258 m/s). The lines are a fit to the data taken at $Q_x$=5*10$^{-2}$ Å$^{-1}$, $Q_y$=6*10$^{-3}$ Å$^{-1}$ using Eq. (1). For definitions of x and y see Fig. 2.

Figure 1 shows a typical measurement of the modulation of the neutron beam polarization projected onto the analyser direction on the NSE instrument at the NIST Center for Neutron Research (NCNR, National Institute for Standards and Technology, Gaithersburg, MD, USA). The period of the oscillations corresponds to one additional spin rotation in one or the other precession coil. The highest polarisation is recovered for identical number of rotations in the first and second precession coil. For any difference in the number of rotations neutron waves of different wavelength do not fully recover their polarisation. Therefore, the echo has a finite width for a finite wavelength distribution in the neutron beam that can be approximated by a Gaussian distribution:

1 $$\begin{aligned} P_{\text {raw}}=A_{0}-A_{\text {raw}}\exp \left( -\frac{(\phi _{\text {raw}}-\phi _0)^2}{2W^2}\right) *\cos ((\phi _{\text {raw}}-\phi _0)/T), \end{aligned}$$

with the average unpolarized beam intensity $A_0$, the raw amplitude $A_{\text {raw}}$, the raw phase $\phi _{\text {raw}}$, the phase offset $\phi _0$ and the period *T*. The sigma width of the wavelength distribution is related to $W = T*\Delta \lambda /\lambda$. In Figure 1 the experimental points for a static graphite block are shown as circles, whereas the solid line is a fit using Eq. (1). The measured width of the echo could be best fitted with a wavelength distribution of $\Delta \lambda /\lambda = 7.3\%$ (sigma), which corresponds well to the 20% full width produced by the velocity selector. A second NSE signal is shown for a sample moving at a constant velocity (triangles) and resulting in inelastic Doppler scattered neutrons. It can be clearly seen that this results in a phase shift of the echo point as well as in a decreased amplitude (depolarisation) due to a non uniform sample velocity.

As the depolarisation from the inelastic Doppler scattering is superimposing with the one from the molecular dynamics the two effects need to be separated. In this paper we measure and calculate the effect of inelastic Doppler scattering on NSE data. We show that the depolarisation and phase from a graphite disc moving at a known speed can be calculated and how beam divergence and a distribution of sample velocities may be taken into account. In the rest of the article we show the phases and amplitudes corrected by a graphite resolution measurement ($\phi =\phi _{\text {raw}}-\phi _0$ and $A=A_0/A_0^{\text {graphite}}$).

## Doppler scattering in NSE

For NSE a shift in neutron speed at the sample will result in a phase shift $\Delta \phi$ of the measured echo point as can be seen by the triangles in Fig. 1. In case of slow sample speeds $v_s\ll v_n$, as is the case here, the frequency shift $\Delta f$ of the neutron wave due to the Doppler effect can be approximated as:

2 $$\begin{aligned} \Delta f = f_0 \frac{v_s}{v_n}, \end{aligned}$$

with the initial frequency of the neutron beam $f_0=\frac{v_n}{\lambda }$. Here $\lambda$ is the wavelength of the neutrons. The phase shift of the echo can be calculated as:

3 $$\begin{aligned} \Delta \phi&= 2\pi \Delta f\tau = v_s\tau \frac{2\pi }{\lambda }. \end{aligned}$$

Here $\tau$ is the observation time or Fourier time and we assume that the sample speed is parallel to the direction of observation. In scattering experiments the observer angle with respect to the incident angle is $2\theta$ or the scattering angle. Therefore the observation direction is along the momentum transfer, $\vec {Q}$, which is the difference between the scattered and incoming wave vectors:

4 $$\begin{aligned} \vec {Q}=\vec {k_f}-\vec {k_i} \text{ and } |Q| = \frac{2\pi }{\lambda }\sin (2\theta ). \end{aligned}$$

For the Doppler shift only the component of the sample speed $\vec {v}$ projected along the direction of $\vec {Q}$ has to be taken into account $\Delta \phi =\vec {Q}\vec {v}\tau$. For a single wavelength and perfectly collimated beam, a point detector and a constant sample velocity over the entire beam cross-section, the shift in phase would not engender any decrease in polarization but only a phase shift of the echo point, which can be compensated by tuning one of the precession coils. For real situations, however, the wavelength spread ($\Delta \lambda$), the angular smearing due to beam divergence and pixel size on the detector ($\Delta \theta _{x,y}$) as well as a spread of sample velocities over the illuminated sample volume will result in a superposition of echos with slightly different phases. This results in an effective depolarisation, which can be calculated by superposition. The final polarization is a function of tuning coil phase $\phi$ and the integral over the illuminated sample area, $2y_0w$, with *w* the width and $2y_0$ the height of the sample slit, the wavelength spread $\Delta \lambda$ and the angular divergence $\Delta \theta _{x,y}$:

5 $$\begin{aligned} P(\phi )=\frac{\int _{\Delta \lambda }\int _{\Delta \theta _{x,y}}\int _{w}\int _{2y_0}\exp (i(\phi +\Delta \phi )) \partial y \partial x \partial \theta _{x,y}\partial \lambda }{\int _{\Delta \lambda }\int _{\Delta \theta _{x,y}}\int _{w}\int _{2y_0} \textbf{1} \partial y \partial x \partial \theta _{x,y}\partial \lambda }. \end{aligned}$$

**Figure 2:**
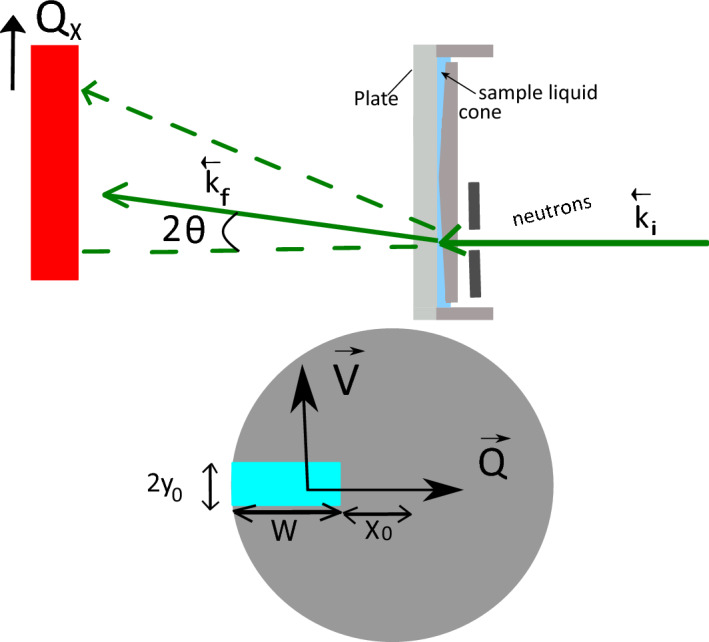
Sketch of the sample and beam geometry. Top panel: Top view of the setup. The neutron beam is transmitted through the cone/plate shear device and registered by a position sensitive detector. Lower panel: Front view of the cone with the neutron window (sample slit) indicated in light blue. The position and the size of the window are marked as well as the directions of the mean values of $\vec {v}$ and $\vec {Q}$.

Note, for the scattering angle the scalar product between $\vec {Q}$ and $\vec {v}$ has been taken into account. The scattering and sample geometry is shown in Fig. 2. We note here as well that only the real part of the neutron beam polarization along the analyzer direction is measured, but in order to simplify the equations we use the complex terminology for the polarization here.

By introducing the mean phase shift:

6 $$\begin{aligned} <\Delta \phi > = \frac{\int _{\Delta \lambda }\int _{\Delta \theta _{x,y}}\int _{w}\int _{2y_0}\vec {Q}\vec {v}\tau \partial y \partial x \partial \theta _{x,y}\partial \lambda }{\int _{\Delta \lambda }\int _{\Delta \theta _{x,y}}\int _{w}\int _{2y_0} \textbf{1} \partial y \partial x \partial \theta _{x,y}\partial \lambda }, \end{aligned}$$

Equation 5 can be restructured to:

7 $$\begin{aligned} P(\phi )= \exp (i(\phi +<\Delta \phi>))A_{\text {eff}} =\exp (i(\phi +<\Delta \phi>))\frac{\int _{\Delta \lambda }\int _{\Delta \theta _{x,y}}\int _{w}\int _{2y_0}\exp (i(\Delta \phi -<\Delta \phi >)) \partial y \partial x \partial \theta _{x,y}\partial \lambda }{\int _{\Delta \lambda }\int _{\Delta \theta _{x,y}}\int _{w}\int _{2y_0} \textbf{1} \partial y \partial x \partial \theta _{x,y}\partial \lambda }, \end{aligned}$$

The first part is the echo, but shifted by the mean phase due to Doppler scattering. The quotient on the right is the amplitude, $A_{\text {eff}}$, of the echo due to the spread in phase shifts resulting from different energy transfers at the sample. For a single wavelength and no angular divergence only the different sample velocities with respect to q need to be taken into account and $A_{\text {eff}}$ can be calculated analytically:

8 $$\begin{aligned} A_{\text {ana}} = \frac{\int _{x_0}^{x_0+w}\int _{y_1-y_0}^{y_1+y_0}\exp (i(\vec {Q}\vec {v}\tau -<\Delta \phi _{\text {ana}}>)) \partial y \partial x }{\int _{x_0}^{x_0+w}\int _{y_1-y_0}^{y_1+y_0}\textbf{1} \partial y \partial x}, \end{aligned}$$

with the mean phase shift:

9 $$\begin{aligned} <\Delta \phi _{\text {ana}}> = \frac{\int _{x_0}^{x_0+w}\int _{y_1-y_0}^{y_1+y_0}\vec {Q}\vec {v}\tau \partial y \partial x }{\int _{x_0}^{x_0+w}\int _{y_1-y_0}^{y_1+y_0}\textbf{1} \partial y \partial x}. \end{aligned}$$

Here *w* is the width of the beam window starting at a distance $x_0$ from the rotation axis. $y_0$ is half of the vertical beam size offset by $y_1$ from the rotation center in vertical direction. Here we assume a constant beam intensity along the sample gap (top-hat beam profile), which is a reasonable assumption if the collimation slit is much larger than the sample slit and the latter is placed very close to the sample. Note that for other beam shapes, e.g. Gaussian beams typically describing laser beams, the amplitude function can be significantly different at large scattering angles^18^.

## Rotating solid block

A solid block of elastic scatterer, e.g. graphite, rotating at the cone position at a constant angular velocity can be described by the velocity vector below:

10 $$\begin{aligned} \vec {v}_{\text {block}}&= \begin{bmatrix} -2y\pi * n \\ 2x\pi * n \\ 0 \end{bmatrix} \end{aligned}$$

with *n* being the rotation speed in rounds per s.

With this velocity profile the mean phase can be calculated analytically:

11 $$\begin{aligned} <\Delta \phi _{\text {ana}}>=\frac{2\pi ^2 n\tau }{\lambda }\left( (2x_0+w)\sin (2\theta _y)-2y_1\sin (2\theta _x)\right) . \end{aligned}$$

And for the amplitude we get:

12 $$\begin{aligned} A_{\text {ana}}=\frac{\sin (2\pi n\tau q_xy_0)*\sin (\pi \tau q_yw)}{2(\pi n\tau )^2q_xq_ywy_0}. \end{aligned}$$

By substituting the scattering angles and the wavelength by the corresponding momentum transfers $q_{x,y}$ using Eq. (4). This function is similar to the decorrelation functions calculated in case of Couette flow in 1D for homodyne light^18^ and X-ray^19^ spectroscopy.

Alternatively, the mean phase (Eq. 6) can be calculated numerically by replacing the integrals by sums and coarse-graining the integrals over the sample slit (in directions *x*, *y*, *z*) and detector pixel (in directions $p_x,p_y$) into *N* points. It turns out that a wavelength spread $\Delta \lambda /\lambda<$ 20%, as typical on NSE machines, is not influencing the result significantly for the parameters considered here. This is due to the fact that the echo amplitude for a 360$^{\circ }$ phase shift only decreases by less than 10% for a 20% full width wavelength spread, as can be seen in Fig. 1, whereas the amplitude decrease due to Doppler scattering for phase shifts of 360$^{\circ }$ are massive (see e.g. Fig. S2 in the Supplementary Information with an amplitude decrease to 0.4 at the lower edge of the detector where the phase shift is about 100$^{\circ }$). Therefore Eq. (6) reduces to:

13 $$\left\langle {\Delta \phi _{{{\text{num}}}} } \right\rangle = \frac{{\sum\nolimits_{{x,y,p_{x} ,p_{y} }}^{N} {\vec{q}} \vec{v}\tau }}{{N^{4} }}.$$

Note that for the numerical solution the illuminated sample volume may be summed over all three dimensions including the direction along the beam (*z*) in order to allow liquid samples with a velocity gradient along *z*. Moreover, as explained above, the sum over the detector pixels is extended beyond the real detector pixel size to include the incoming beam divergence.

In order to calculate the effective amplitude Eq. (7) can be solved numerically. This requires *a priori* knowledge of the mean phase and is thus computationally slow. Instead we calculate the *x* and *y* projections of $\vec {q}$ and $\vec {v}$ and sum them over the sample volume and detector pixel divided into N points similar to the numerical mean phase calculation. The root of the summed squares gives the effective amplitude:

14 $$\begin{aligned} A_{\text {num}} = \sqrt{ \frac{\left( \sum \nolimits _{x,y,p_x,p_y}^{N}\sin (\vec {q}\vec {v}\tau )\right) ^2 + \left( \sum \nolimits _{x,y,p_x,p_y}^{N}\cos (\vec {q}\vec {v}\tau )\right) ^2}{N^{8}}} \end{aligned}$$

This calculation is equivalent to estimating the absolute length of the vector resulting by summing up all vectors with an angle equivalent to the phase $\phi$ within the *x*, *y*-plane.

**Figure 3:**
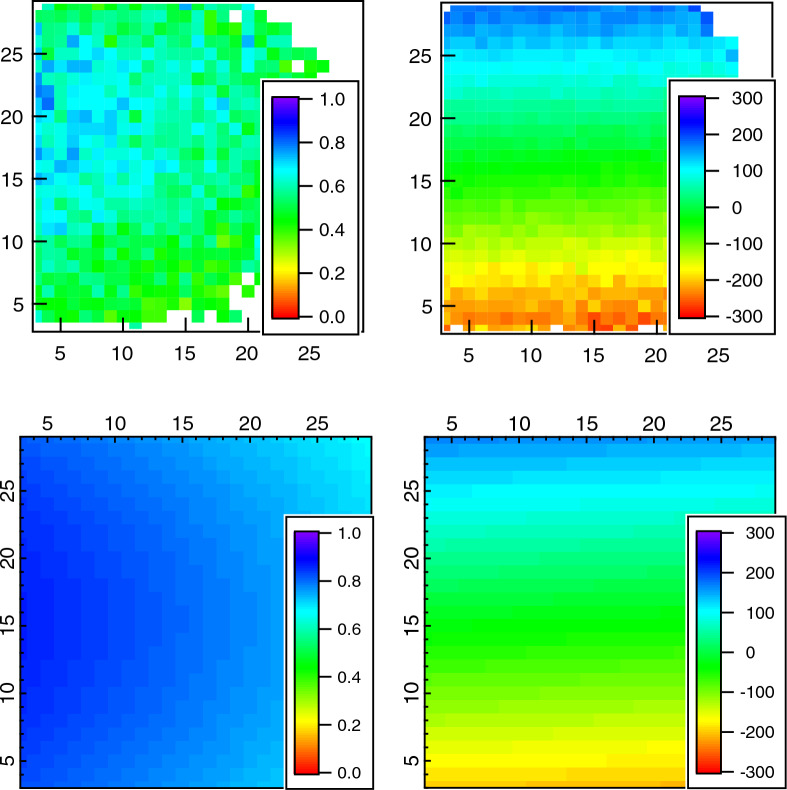
(Top panel experimental, bottom simulation) 2D phase (right panel) and amplitude (left panel) images at a detector angle of 6$^\circ$ and a Fourier time of $\tau =141$ ns from a graphite block rotating at a speed of 73 rpm. Note that the amplitude maps have not been corrected for beam divergence, hence the overestimation of the amplitude. This is done in Fig. 4 Right.

To validate the numerical simulations (Eq. 14) we compare the calculated phases and amplitudes for a rotating solid sample (velocity profile from Eq. 10) to the analytical approximation (Eq. 12) by assuming no beam divergence. In addition we compare to experimental data from a rotating graphite spiral normalized to the same graphite at rest. This data was measured using a shear device described elsewhere^20^. The calculated and measured amplitude and phase are shown in the contour plots in Fig. 3. The qualitative agreement is excellent.

**Figure 4:**
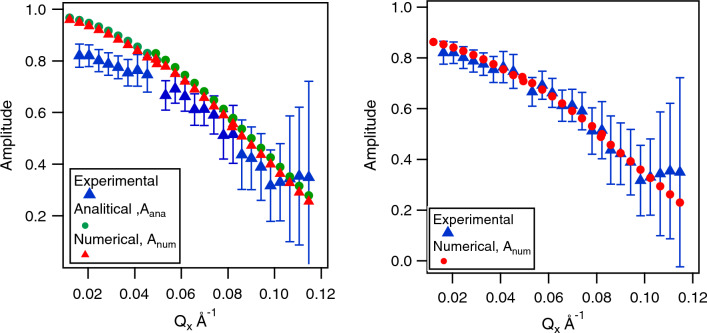
(left panel) Experimental amplitudes as a function of detector angle for the data from Fig. 3 for Fourier time of 141 ns from a graphite block rotating at a speed of 73 rpm. For the numerical simulations represented in the right panel a beam divergence of 0.2$^{\circ }$ has been added.

For a more quantitative analysis Fig. 4 depicts the measured effective amplitudes plotted as a function of $Q_x$ for $Q_y=0$ (averaging the signal over three detector pixels in *y* direction corresponding 0.003 Å$^{-1}$ in Q range).

**Figure 5:**
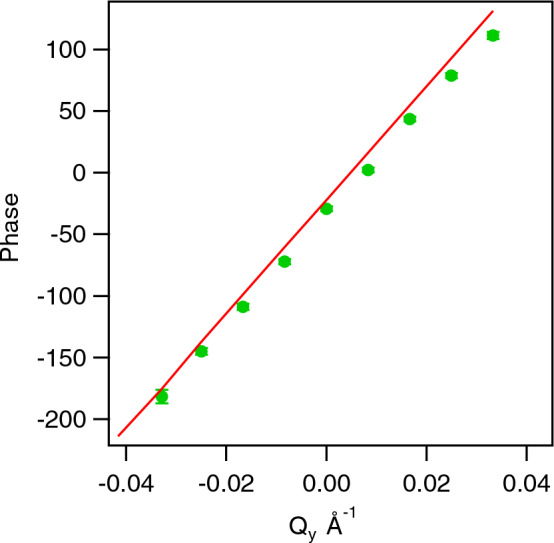
Experimental (circles) and numerically calculated (line) phase as a function of vertical detector angle for the data from a rotating solid block at a speed of rotation of 73 rpm and a Fourier time of 141 ns and a horizontal detector angle of 6$^{\circ }$.

Data is shown for a Fourier time of 141 ns. The analytic and numerical calculations agree very well. However, the experimental data clearly show a stronger depolarisation, in particular, at low $\vec {Q}$ values. This discrepancy can be resolved by adding angular beam divergence to our simulations by artificially expanding the integration range of the detector pixel. By increasing the effective divergence in the outgoing beam we can accommodate for the lack of divergence in the incident beam. Fig. 4 shows a perfect match of the numerical simulation with the experimental data assuming a beam divergence of 0.2$^{\circ }$ (1$\sigma$), which is slightly larger, but close to the theoretical instrumental beam divergence of 0.15$^{\circ }$ (FWHM). This confirms that including the beam divergence is sufficient to accurately describe the Doppler scattering. We note here that the scattered beam divergence due to a finite detector pixel size is about 0.06$^{\circ }$ (1$\sigma$) on both spectrometers used and is therefore significantly smaller, compared to the incoming beam divergence. Our calculations show that the phase and amplitude changes due to Doppler scattering can be determined exactly. Figure 5 shows the calculated and experimentally determined phase shift along $Q_y$, which is parallel to $\vec {v}$.

Again, our calculations are in excellent agreements with the measured data.

These calculations open the possibility to enforce the calculated phase in the raw 2D data treatment of detector images in order to improve the quality of the extracted amplitudes and normalise the recorded amplitudes by the simulated maps to correctly account for the Doppler scattering from a moving sample.

## Sheared liquid sample

In case of a liquid sheared between a plate and a cone with an angle $\alpha$, as depicted in Fig. 2, the velocity depends on the position along z, i.e. along the beam:

15 $$\begin{aligned} \vec {v}_{\text {liquid}}&= \begin{bmatrix} \frac{-2yz\pi n}{\tan {(\alpha )}\sqrt{x^2+y^2}} \\ \frac{2xz\pi n}{\tan {(\alpha )}\sqrt{x^2+y^2}} \\ 0 \end{bmatrix} \end{aligned}$$

Therefore the integrals of the mean phase and the effective amplitude have to include the integral along z:

16 $$\begin{aligned} <\Delta \phi >= & {} \frac{\int _{\Delta \lambda }\int _{\Delta \theta _{x,y}}\int _{w}\int _{2y_0}\int _{z=0}^{\tan {(\alpha )}\sqrt{x^2+y^2}}\vec {q}\vec {v}\tau \partial y \partial x \partial z \partial \theta _{x,y}\partial \lambda }{\int _{\Delta \lambda }\int _{\Delta \theta _{x,y}}\int _{w}\int _{2y_0}\int _{z=0}^{\tan {(\alpha )}\sqrt{x^2+y^2}} \textbf{1} \partial y \partial x \partial z \partial \theta _{x,y}\partial \lambda } \end{aligned}$$

17 $$\begin{aligned} A_{\text {eff}}= & {} \frac{\int _{\Delta \lambda }\int _{\Delta \theta _{x,y}}\int _{w}\int _{2y_0}\int _{z=0}^{\tan {(\alpha )}\sqrt{x^2+y^2}}\exp (i(\vec {q}\vec {v}\tau -<\Delta \phi >)) \partial y \partial x \partial z \partial \theta _{x,y}\partial \lambda }{\int _{\Delta \lambda }\int _{\Delta \theta _{x,y}}\int _{w}\int _{2y_0}\int _{z=0}^{\tan {(\alpha )}\sqrt{x^2+y^2}} \textbf{1} \partial y \partial x \partial z \partial \theta _{x,y}\partial \lambda }. \end{aligned}$$

In order to simplify these calculations for the numerical solution as done in Eqs. (13 and 14) we replace:

18 $$\begin{aligned} z \Rightarrow z' = \frac{z}{\tan {(\alpha )}\sqrt{x^2+y^2}} \end{aligned}$$

and the integration in $z'$ direction is replaced by a sum in steps of $\Delta z' = \frac{\sqrt{x^2+y^2}\tan {(\alpha )}}{<\sqrt{x^2+y^2}>\tan {(\alpha )}N}$,

which results in:

19 $$\begin{aligned}<\Delta \phi _{\text {num}}> = \frac{\sum \nolimits _{z'=0}^{N}\sum \nolimits _{x,y,p_x,p_y}^{N}\vec {q}\vec {v}\tau * \sqrt{x^2+y^2} }{<\sqrt{x^2+y^2}>N^5} \end{aligned}$$

and

20 $$\begin{aligned} A_{\text {num}} = \frac{\sqrt{\left( \sum \nolimits _{z'=0}^{N}\sum \nolimits _{x,y,p_x,p_y}^{N}\sin (\vec {q}\vec {v}\tau )* \sqrt{x^2+y^2}\right) ^2 + \left( \sum \nolimits _{z'=0}^{N}\sum \nolimits _{x,y,p_x,p_y}^{N}\cos (\vec {q}\vec {v}\tau )* \sqrt{x^2+y^2}\right) ^2}}{<\sqrt{x^2+y^2}>N^{5}} \end{aligned}$$

**Figure 6:**
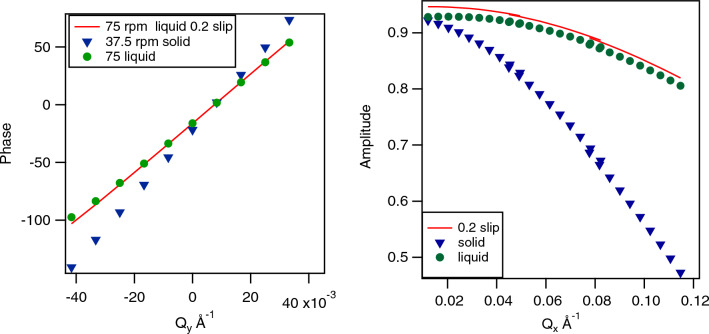
Left panel: Numerically calculated phases as a function of vertical detector angle (along $Q_y$) for a solid block using Eq. (13) and a speed of rotation of 37.5 rpm (triangles) and for a liquid sample using Eq. (19) at a cone rotation speed of 75 rpm, with a linear velocity profile, leading to the same average speed (circles). The solid line is for a liquid sample at 75 rpm using the same parameters but with a speed deviation of 20% at the shearing surfaces, simulating massive symmetric slip. Right panel: Numerically calculated amplitudes as a function of horizontal scattering angle, along $Q_x$, for the same velocity distributions.

The phase calculated with Eq. (19), assuming a linear velocity profile, is shown in Fig. 6 Left panel. Calculations are done for the same parameters as for the graphite disc at a Fourier time of 141 ns, but for twice the rotation speed in order to have the same average speed of the liquid. Clear differences are visible testifying the sensitivity of the spin-echo phase to the velocity profile in general. The Doppler effect on the depolarisation is significantly stronger for the solid disk as compared to the sheared liquid (see Fig. 6 Right Panel) for the same average rotation speed. This imposes limitations to the data normalisation of liquid samples by a rotating solid disc as used previously^20,21^. On the other hand, by normalising to the calculated depolarization as presented here, NSE measurements at higher rotation speeds and longer Fourier times should become possible. In order to evaluate the influence of more subtle changes in the shear profile, as for example surface slip, which may be present in entangled polymer solutions, we simulated in addition the phase and amplitude maps assuming a massif symmetric slip scenario, assuming a reduction of the liquid speed by 20% at the moving surface and symmetrically 20% of the rotation speed in the liquid at the stationary surface. This scenario is called 0.2 slip in Fig. 6. As can be seen the different liquid velocity profiles do not show any significant change in the mean phase along $Q_y$, as the mean liquid speed is the same limiting the sensitivity of the NSE phase to subtle velocity profile changes. The amplitude, however, shows an almost constant offset along $Q_x$, testifying the need to take into account the exact velocity profile for the here proposed Doppler normalization. However, we note that this signature is different from any expected changes in the microscopic dynamics of a liquid or polymer under shear and thus it should be possible to distinguish between changes in microscopic dynamics under shear and different shear profiles due to flow instabilities for example.

## Implications for Rheo-NSE

The position of the sample slit has no influence on the depolarization for a solid block, but for a liquid sample the depolarization is increased if the center of the slit is further away from the cone center due to the increased sample thickness and thus the velocity spread along the beam. Therefore it is advisable to place the slit as close to the center as possible, at least for the y-direction in Fig. 2. In the x-direction the slit should be close to the center as well, but avoiding the truncated part of the cone. It has to be noted, though, that moving the slit closer to the center in x-direction means a smaller sample volume in case of cone/plate geometry and thus less scattered intensity, so a compromise might be needed for low scattering samples. As expected the width of the sample slit has a lesser effect on depolarization as the height, making a slit shaped beam ideal for these experiments as shown in Fig. 2. Smaller beam divergence reduces the de-polarization at small momentum transfers, but given the high echo amplitude for these small angles in general, and the usually big gain in neutron flux if increasing beam divergence, it is considered not worth optimising this parameter. The same is true for the neutron wavelength and wavelength resolution: both have negligible influence on the Doppler-induced depolarisation at constant momentum transfer and thus the usual settings on NSE machines involving a big range of wavelengths (as on time-of-flight machines) and a large wavelength spread (typically $\Delta \lambda /\lambda =20\%$ on monochromatic machines) is compatible with *in situ* shear - NSE.

Concerning the best measurement strategy it is advisable to first determine all experimental parameters, such as effective sample slit size and position as well as beam divergence by using a strongly elastic scattering graphite block as done here. Next, in order to optimise the measurement one could imagine to compensate actively the expected phase shift during the measurement with the magnetic field coils of the instrument. This would also eliminate any influence of wavelength spread on beam depolarization. Consequently, the only “normalisation” step left would be to divide the measured spin amplitudes by the calculated Doppler-reduced values.

## Methods

The neutron spin echo experiments were performed on the monochromatic spectrometer IN15^22^ at the Institut Laue-Langevin (ILL), Grenoble, France. The wavelength was fixed to 10 Å  with a wavelength resolution of 18% (FWHM). The beam footprint on the sample was defined by a sample slit set to 4$\times$8 mm$^2$. The beam divergence was limited by a 10 mm slit, 3.6 m away from the sample. The sample was contained in a recently developed shear cell described elsewhere^20^. The 2D detector with 32$\times$32 pixels with a size of $10\times 10$ mm$^2$ was placed 4780 mm away from the sample. The echo for each Fourier time was measured determining the polarization at four tuning coil phases and using an automatic fitting method implemented in IgorPro. All raw 2D detector images were normalized to a static graphite spiral (3 mm thick) measurement on a pixel-by-pixel basis. Doppler scattering was measured by rotating the same disc at a constant angular velocity. The neutron spin echo experiments were also performed on the spectrometer located on the end position of neutron guide A (NG-A) at the National Institute of Standards and Technology (NIST) Center for Neutron Research (NCNR). The incoming wavelength was fixed to 11 A with a wavelength resolution of 20%. A similar sample setup to the one employed at ILL was used. The 2D detector with 32$\times$32 pixels with a size of $10\times 10$ mm$^2$ was placed 4.35 m away from the sample. The software Dave was used for the data reduction.

### Supplementary Information

Supplementary Information.

## Data Availability

The datasets used and/or analyzed during the current study are available from the corresponding author upon reasonable request.
