# Supplementary material for: Neutron spin echo spectroscopy with a moving sample
Source: Sci Rep. 2023 Aug 11;13:13051. doi: 10.1038/s41598-023-39854-4 (PMC10421866; doi:10.1038/s41598-023-39854-4)
Supplement: Supplementary file 1 — Supplementary Information. [file 41598_2023_39854_MOESM1_ESM.pdf]

Supplementary Information: Neutron spin echo spectroscopy with a moving sample

Manuchar Gvaramia[1,+], Philipp Gutfreund[2,+,*], Peter Falus[2], Antonio Faraone[3], Michihiro Nagao[3,4,5], and Max Wolff[1,*]

[1]Department for Physics and Astronomy, Uppsala University, Regementsvägen 1, SE-75120 Uppsala, Sweden
[2]Institut Laue–Langevin, CS 20156, 38042 Grenoble Cedex 9, France
[3]Center for Neutron Research, National Institute of Standards and Technology, Gaithersburg, MD, United States
[4]Department of Materials Science and Engineering, University of Maryland, College Park, MD, United States
[5]Department of Physics and Astronomy, University of Delaware, Newark, DE, United States
[*]corresponding. gutfreund@ill.eu, max.wolff@physics.uu.se
[+]these authors contributed equally to this work

ABSTRACT

Additional Data.

Figure S1 depicts calculated and measured amplitudes for the same experimental conditions as shown in Fig. 4 in the main article but for shorter Fourier times of 5 ns and 42.5 ns.

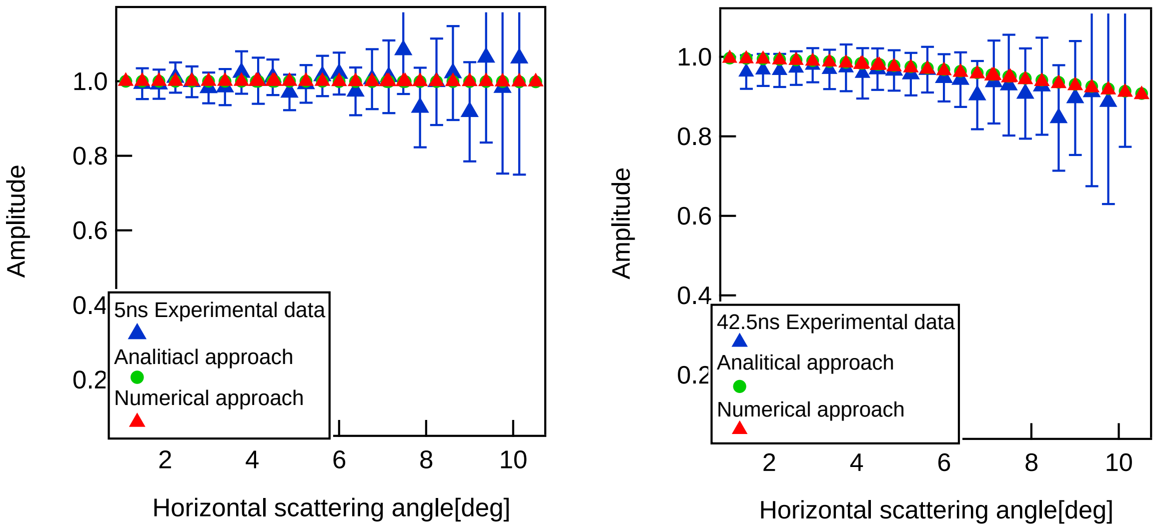

Figure S1. Experimental amplitudes as a function of detector angle for Fourier times of (a) 5 ns, (b) 42.5 ns from a graphite block rotating at a speed of 73 rpm.

Finally, the 2D amplitude map for a liquid sample at 75 rpm without slip using the same parameters as in Fig. 6 of the main text is shown in Fig. S2.

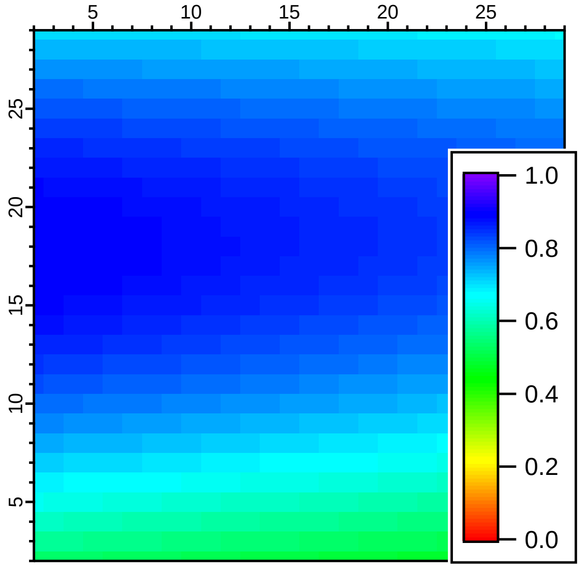

Figure S2. Numerically calculated 2D amplitude for a liquid sample at 75 rpm without slip using the same parameters as in Fig. 6 of the main text at a detector angle of 9°.
